# *Listeria monocytogenes* Internalin B Activates Junctional Endocytosis to Accelerate Intestinal Invasion

**DOI:** 10.1371/journal.ppat.1000900

**Published:** 2010-05-13

**Authors:** Mickey Pentecost, Jyothi Kumaran, Partho Ghosh, Manuel R. Amieva

**Affiliations:** 1 Department of Microbiology and Immunology, Stanford University, Stanford, California, United States of America; 2 Department of Chemistry and Biochemistry, University of California San Diego, La Jolla, California, United States of America; 3 Department of Pediatrics, Stanford University, Stanford, California, United States of America; Institut Pasteur, France

## Abstract

*Listeria monocytogenes* (*Lm*) uses InlA to invade the tips of the intestinal villi, a location at which cell extrusion generates a transient defect in epithelial polarity that exposes the receptor for InlA, E-cadherin, on the cell surface. As the dying cell is removed from the epithelium, the surrounding cells reorganize to form a multicellular junction (MCJ) that *Lm* exploits to find its basolateral receptor and invade. By examining individual infected villi using 3D-confocal imaging, we uncovered a novel role for the second major invasin, InlB, during invasion of the intestine. We infected mice intragastrically with isogenic strains of *Lm* that express or lack InlB and that have a modified InlA capable of binding murine E-cadherin and found that *Lm* lacking InlB invade the same number of villi but have decreased numbers of bacteria within each infected villus tip. We studied the mechanism of InlB action at the MCJs of polarized MDCK monolayers and find that InlB does not act as an adhesin, but instead accelerates bacterial internalization after attachment. InlB locally activates its receptor, c-Met, and increases endocytosis of junctional components, including E-cadherin. We show that MCJs are naturally more endocytic than other sites of the apical membrane, that endocytosis and *Lm* invasion of MCJs depends on functional dynamin, and that c-Met activation by soluble InlB or hepatocyte growth factor (HGF) increases MCJ endocytosis. Also, *in vivo*, InlB applied through the intestinal lumen increases endocytosis at the villus tips. Our findings demonstrate a two-step mechanism of synergy between *Lm*'s invasins: InlA provides the specificity of *Lm* adhesion to MCJs at the villus tips and InlB locally activates c-Met to accelerate junctional endocytosis and bacterial invasion of the intestine.

## Author Summary

The anatomical context in which attachment and invasion factors find host receptors determines when and where microbes can colonize and invade. For example, *Listeria monocytogenes* (*Lm*), a cause of human and animal food-borne disease, invades the villous epithelium only at the intestinal villus tips where dying cells are extruded from the epithelium. This is because *Lm's* receptor, E-cadherin, a cell-cell junction protein normally hidden from the intestinal lumen, becomes transiently exposed during the dramatic junctional disassembly and reorganization required for extrusion and maintenance of epithelial continuity. Here, we find that basolateral c-Met, a receptor tyrosine kinase used by *Lm* for invasion of cells in tissue culture, is also activated as a consequence of its exposure on the apical side at cell extrusion sites. *Lm* stimulates c-Met signaling once bacteria have attached to junctional E-cadherin. Furthermore, c-Met activation at cell extrusion sites induces uptake of E-cadherin, accelerating invasion of *Lm*. Thus, *Lm* not only utilizes the dynamic nature of junctional remodeling to attach to villus tips, but also hijacks signaling that controls junctional endocytosis as a mechanism of intestinal barrier breach. Other enteric microbes whose receptors are inaccessible from the lumen may also target remodeling junctions for attachment and invasion.

## Introduction

*Listeria monocytogenes* (*Lm*) is a potentially deadly food-borne pathogen that colonizes the gastrointestinal tract of several mammalian species, and can also cause invasive disease and systemic spread if it crosses the intestinal epithelial barrier [1]. *Lm* evolved two major molecular invasion proteins, referred to here as invasins: Internalin A (InlA, Internalin) and Internalin B (InlB) [2], [3]. These proteins promote internalization into nonphagocytic cells where *Lm* can grow in the cytosol as a facultative intracellular pathogen and directly spread to neighboring cells through actin-based motility [2]–[5]. Listerial invasion of the gastrointestinal tract requires InlA since deletion of the *inlA* gene makes *Lm* avirulent when given through the enteric route [6]. By contrast, *inlA* is dispensable for simulation of late-stage pathogenesis when bacteria are administered intravenously [6]. InlA binds the most distal extracellular domain of E-cadherin, a transmembrane epithelial cell-cell junction protein [7]–[9]. InlB, the second *Lm* surface protein involved in invasion, binds c-Met, a receptor tyrosine kinase (RTK) and the natural receptor for Hepatocyte Growth Factor (HGF) [2], [10]. InlB promotes invasion of multiple mammalian cell types, and has been implicated in liver colonization after intravenous infection of mice [2], [10]–[24]. Although InlB is not essential for fetoplacental infection, it was recently shown to act synergistically with InlA to promote fetoplacental infection of intravenously inoculated pregnant gerbils and transgenic mice expressing a humanized E-cadherin [20], [25], [26]. InlB is also known to function synergistically with InlA during invasion of cultured epithelial cells through an unknown mechanism [2], [13], [20], [24], [27]–[30]. Paradoxically, neither E-cadherin or c-Met are available on the apical or lumenal side of epithelia, thus it was puzzling to understand where *Lm* finds its receptors for invasion of the intestine [31]–[33].

We identified the cell extrusion zone at the tips of the intestinal villi as a novel site for gastrointestinal invasion where *Lm* uses InlA to bind E-cadherin for attachment and entry [30]. The intestinal epithelium is in a constant state of rapid renewal in a process that begins with stem cell division within the crypts, followed by maturation and migration of cells up to the tips of the intestinal villi. Once the oldest cells reach the villus tip, programmed cell death is triggered and individual dying cells are extruded into the lumen [34], [35]. It has been estimated that 1400 cells are shed from each villus tip per day, which is ∼10^11^ cells per day from the human small intestine [34]. Surprisingly, this occurs without disruption of epithelial continuity because the surrounding cells constrict the dying cell and meet to form a new multicellular junction (MCJ) below the extruding cell [30], [35]–[38]. In the process, the cells that form the MCJ may also remove and recycle the old junctions and adhesive contacts by endocytosis [35]. We showed that *Lm* takes advantage of extrusion for adhesion and invasion because MCJs transiently expose basolateral E-cadherin to the lumen of the intestine and at analogous sites in tissue culture [30]. Although a reasonable hypothesis, it is not known whether other basolateral proteins, like c-Met, are exposed to the apical side at MCJs.

In contrast to what has been observed during infection of cultured cells, a role for InlB in the intestinal phase of infection could not be demonstrated previously [20], [24]. However, several observations suggest that InlA and InlB may both function during infection of the gastrointestinal tract. First, the *inlB* gene is immediately downstream of *inlA* and is translated bicistronically with *inlA* [3]. The *inlAB* operon is upregulated when bacteria are in the intestinal lumen or under conditions simulating the gastrointestinal environment, indicating that InlB expression is temporally upregulated prior to bacterial invasion of intestinal tissue [39]–[42]. Finally, InlB promotes invasion of isolated intestinal epithelial cells when InlA-E-cadherin interactions are functional [20]. Thus, we hypothesized that InlB functions synergistically with InlA to promote *Lm* invasion of MCJs of the villus tip extrusion zone and that c-Met may be exposed to lumenal surfaces during cell extrusion.

Until recently, it was not technically feasible to study the functions of InlA and InlB together in commonly utilized animal models since both proteins are ‘species specific’: InlA binds rabbit and guinea pig E-cadherin, but not rat and mouse E-cadherin; InlB activates mouse c-Met, but not guinea pig or rabbit c-Met [24]. The mouse is the predominant animal model for studying systemic Listeriosis and host immune responses following intraperitoneal or intravascular infection [43]. However, an understanding of the intestinal phase of infection has lagged behind, since mice are very resistant to enteric infection with *Lm* due to the absence of the InlA-E-cadherin interaction [6], [9]. To study the intestinal phase of Listeriosis in the mouse, one strategy has been to develop transgenic mice that express a permissive E-cadherin [6], [20]. Alternately, InlA was recently engineered to bind murine E-cadherin (InlA^m^) and is sufficient to reconstitute intestinal invasion after intragastric infection of mice [44], [45].

In this study we constructed *Lm* strains that express InlA^m^ with or without InlB to dissect the role of InlB in a mouse model of enteric infection. We also made strains that express green fluorescent protein (GFP) in order to perform co-infection studies where two strains that are differentially marked are mixed and inoculated together. Using this method we confirm that InlA is essential to invade the extrusion zone of the intestinal villus tips after oral infection, and establish a role for InlB working synergistically with InlA in colonization of the intestinal villi.

Based on published cell biological experiments in non-polarized epithelial cells, we considered three nonexclusive hypotheses for InlB action at MCJs of villus tips. One is that InlB acts directly as an adhesion protein (adhesin) to promote *Lm* uptake, as suggested by experiments with endothelial cells [14]. A second is that InlB activates c-Met to promote cell-cell dissociation, as seen with recombinant HGF or InlB applied to small islands of cultured epithelial cells, thereby allowing access of *Lm* to E-cadherin at the basolateral surface [10]. Finally, we considered that InlB might promote *Lm* invasion by increasing endocytosis of junctional E-cadherin through c-Met activation as shown for HGF action on nonpolarized cells [46], [47]. To study these possibilities on polarized epithelia we used Madin-Darby canine kidney (MDCK) cell monolayers grown on Transwell filters, a well-characterized model epithelium that is permissive for all aspects of the *Listeria*'s intracellular life-cycle including InlA- and InlB-mediated invasion [30], [48]. We discovered that InlB promotes invasion of the MCJs in polarized MDCK monolayers, but not by acting as an adhesin or increasing *Lm* attachment to E-cadherin across the junctions. Instead we find that InlB locally activates c-Met from the lumenal side to modulate the kinetics of invasion. Using endocytosis assays combined with confocal microscopy analysis, we show that both MCJs in tissue culture and the villus tip extrusion zone are naturally more endocytic than other regions of the epithelium and that InlB modulates this process. We propose that *Lm* has evolved a two-step mechanism to hijack and alter junctional remodeling for epithelial attachment and invasion. First *Lm* specifically target and adhere to the MCJs of the villus tips through apically exposed E-cadherin, and then they use InlB to accelerate the recycling of junction components to increase invasion at MCJs.

## Results

### InlB Promotes Invasion of the Villus Tip Extrusion Zone

In order to study InlB and InlA in the same animal model we had to overcome the species specificity of each molecule. We chose to use an InlA mutation that is capable of binding murine E-cadherin (InlA^m^) [44]. In contrast to *Lm* expressing wild type InlA, *Lm* expressing InlA^m^ are pathogenic to mice by enteric inoculation [44]. In the small intestine, InlA^m^ promotes invasion through villous tissue but has no effect on passive bacterial uptake by Peyer's Patches [44]. We infected mice intragastrically with *Lm* that express InlA^m^ and GFP (WT^m^ GFP) to study *Lm* invasion of the intestinal villous epithelium. By culturing fecal pellets at different times after infection, we noted that peak shedding of the inoculum occurs by 3 hours. We therefore chose to examine the small intestine for evidence of bacterial invasion by direct visualization of tissue whole mounts within 4–6 h of infection.

**Figure 1:**
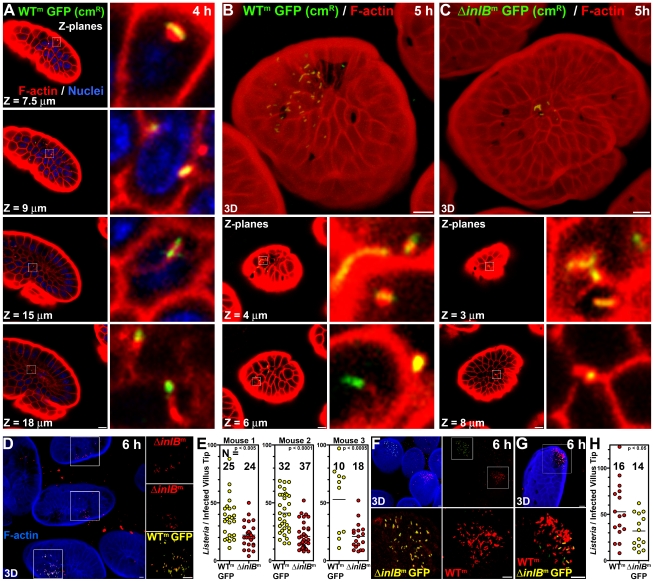
InlB-mediates invasion of intestinal villus tips. (A) Confocal Z-planes of an ileal villus tip from a mouse infected with 10^10^ CFU WT^m^ GFP for 4 h and counterstained for F-actin, red, and nuclei, blue. Insets show intracellular *Lm* with associated F-actin. Depth from the apical cell surface, Z, is indicated. (B) Top panel, a 3D confocal reconstruction of an ileal villus tip of a mouse infected with 10^10^ CFU WT^m^ GFP for 5 h and counterstained for F-actin, red. Lower panels, Z-planes and insets of intracellular *Lm* with associated F-actin. (C) Top panel, a 3D confocal reconstruction of an ileal villus tip of a mouse infected with 10^10^ CFU Δ*inlB* ^m^ GFP for 5 h and counterstained for F-actin, red. Lower panels, Z-planes and insets of intracellular *Lm* associated with F-actin. (D–E) Coinfection with 5×10^9^ CFU each WT^m^ GFP and Δ*inlB* ^m^ *Lm* for 6 h. Tissue was stained with phalloidin for F-actin, blue, and for all *Lm*, red. (D) 3D confocal reconstruction of infected villus tips. (E) Quantification of *Listeria* per infected villus tip for 3 mice. (F–H) Coinfection with 5×10^9^ CFU each WT^m^ and Δ*inlB* ^m^ GFP *Lm* for 6 h. Tissue was stained with phalloidin for F-actin, blue, and for all *Listeria*, red. (F) Top left, 3D confocal reconstruction of infected villus tips. Top right, F-actin staining from the top left panel is omitted to show all *Lm*. Bottom panels, zoomed insets from top right. (G) A rare villus tip infected with both WT^m^ and Δ*inlB* ^m^ GFP *Lm*. (H) Quantification of *Listeria* per infected villus tip. Scale bars, 10 µm.

We find that WT^m^ GFP invade the extrusion zone at the tips of the murine intestinal villi, similar to what we previously reported for *Lm* in a rabbit ileal loop model, and in accord with the observation by Wollert et al. that a similarly modified strain invades the murine intestinal epithelium [30], [44]. Infected villus tips were most abundantly observed in tissue from the terminal ileum, in agreement with previous observations of enteric infection of permissive animals [49], [50]. We found that that ingestion of *Lm* does not result in generalized invasion of all intestinal villi. Rather, we find that infection occurs at sporadic villus tips (Figure 1). We used 3D confocal microscopy analysis to characterize *Lm* invasion of villus tips within ∼1 cm^2^ tissue sections from the terminal ileum (Figure 1, Figure S1, Video S1). Intracellular *Lm* with polymerized actin comet tails are observed in villus tips by 4 hours after intragastric inoculation (Figure 1A), only slightly longer than the time needed to generate actin-based motility in tissue culture (∼3 h) [30], [48], [51]. Thus *Lm* rapidly traffic through the murine bowel and establish initial infection of villus tips.

To examine the role of InlB in intestinal infection, we inoculated mice with either WT^m^ GFP or an isogenic strain lacking *inlB* (Δ*inlB* ^m^ GFP) and examined the small intestine using confocal microscopy to determine the frequency of infected villi and the number of *Listeria* per infected villus tip. Both strains preferentially invade the terminal ileum and invade approximately the same number of villi (N) within a section of tissue by 5 hours post inoculation (Figure S1). However, mice infected with WT^m^ GFP have approximately twice the number of *Lm* per villus tip than mice infected with Δ*inlB* ^m^ GFP (Figure 1B–C, 3D rendered top panels, Figure S1). Both strains are able to escape the endosome and replicate in the cytosol of enterocytes since they induce actin polymerization on the bacterial surface, as observed in Z-planes located below the apical brush border (Figure 1B–C, lower panels). To control for variability between mice in intestinal transit, and thus more stringently examine whether InlB is involved in early colonization of the villus tips, we mixed the two strains at a 1∶1 ratio and performed co-infection experiments. In order to distinguish the two strains, we tagged them differentially with GFP and then counterstained them with anti-*L. monocytogenes* antibodies in red. Thus, the GFP expressing strain appears yellow (or a combination of red and green) in a merged image and the non-GFP expressing strain appears red (Figure 1D–H).

As shown in Figure 1D, in co-infections with WT^m^ GFP and Δ*inlB* ^m^, scattered villi are infected. In all co-infections, the villus tips infected with WT^m^ GFP have significantly more intracellular bacteria than villus tips infected with Δ*inlB* ^m^ at 6 hours, even though the number of infected villi by each strain (N) was similar (Figure 1E). We switched the strains in which GFP was expressed to control for possible variations in antibody staining or possible effects of GFP on bacterial colonization (Figure 1F–H). As with the converse experiment, the presence of *inlB* significantly increases villus tip infection (Figure 1H). The majority of bacterial plaques within each infected villus are probably clonal since we found only 1 villus tip with both red and yellow bacteria (Figure 1G) among 175 infected villi analyzed (Figure 1E, 1H).

### InlB Accelerates Apical Invasion at Multicellular Junctions but Does Not Act as an Adhesin

**Figure 2:**
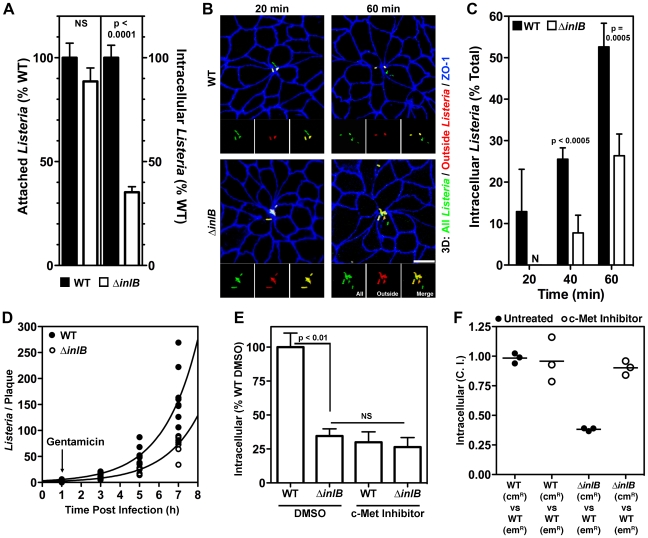
InlB and c-Met accelerate invasion of multicellular junctions. (A) Confluent MDCK monolayers were infected with WT or Δ*inlB Lm* at an MOI of 100. Adhesion after 10 min of infection determined by quantification of all cell-associated CFUs, left, or invasion determined by quantification of viable CFUs of intracellular bacteria after gentamicin treatment, right. Mean and SD from a representative experiment performed in triplicate is shown. (B) Polarized MDCK monolayers were infected with an MOI of 10 of GFP-expressing *Lm*. Invasion at multicellular junctions was visualized with anti-ZO-1 antibodies, blue. To evaluate intracellular versus extracellular bacteria, extracellular adherent GFP-expressing *Lm* were stained before permeabilization in red. External *Lm* thus appear as a combination of red/green or yellow. Scale bars 10 µm. (C) Quantification of intracellular bacteria from monolayers as in B. Mean and SD from three 60X fields are shown. (D) Polarized MDCK monolayers infected with an MOI of 10 were fixed at the indicated time points post infection and the number of *Listeria* per plaque quantified by confocal immunofluorescence analysis. Exponential curves were fit to all data points per strain. WT T_d_ = 1.25 h; R^2^ = 0.818. Δ*inlB* T_d_ = 1.26 h; R^2^ = 0.898. (E) Confluent MDCK monolayers were treated with either DMSO or a c-Met inhibitor prior to infection with WT or Δ*inlB Lm* at an MOI 100 and quantification of viable CFUs of intracellular bacteria after gentamicin treatment. Mean and SD from triplicate samples are shown. (F) To determine whether the invasion defect of Δ*inlB* could be rescued, confluent MDCK monolayers were either untreated or treated with c-Met inhibitor prior and during infection with a 1∶1 ratio of WT∶WT, as a control, or WT∶Δ*inlB* at an MOI of 100. The ratio of the strains, competitive index (C.I.), recovered after gentamicin treatment was determined.

To better understand how InlB promotes invasion of the villus tip extrusion zone, we studied the kinetics and mechanisms of *Lm* invasion in polarized epithelial cells (Figure 2). We used MDCK cells grown on Transwell supports to visualize and study events at multicellular junctions (MCJs). Several clues of InlB function have been derived from studies using recombinant InlB, a genetically modified InlB that is covalently linked to the bacterial cell wall (InlB-SPA), or InlB-coated beads interacting with non-polarized epithelia [10], [11], [19], [24], [52]–[61]. These studies indicate that InlB can bind and activate the basolateral c-Met receptor leading to clathrin-mediated internalization of c-Met. It is not known whether InlB functions for *Lm* invasion as a soluble or a bacterium-associated factor or how InlB reaches this receptor in an intact epithelium since c-Met is not usually exposed on the apical membrane of polarized epithelia [62], [63]. Additionally, there are conflicting data regarding the role of InlB in intracellular replication [64], [65].

We infected MDCK monolayers polarized on Transwell filters from the apical side with GFP-expressing wild type *Lm* (WT) or GFP-expressing *inlB*-mutant *Lm* (Δ*inlB*) and analyzed attachment, invasion and intracellular replication. Attachment to the apical surface is not affected by the absence of InlB as determined by recovered colony forming units (CFUs) from a 10-minute attachment assay (Figure 2A). This is in agreement with our previous finding that InlA, rather than InlB, is the dominant adhesin for polarized cells [30]. Microscopic examination of the sites of attachment shows that Δ*inlB* also bind exclusively at intercellular junctions and preferentially at MCJs with the same specificity and frequency as WT ([30] and see below).

Since attachment was not affected by InlB, we studied its role in invasion following attachment by incubating adhered WT or Δ*inlB* with the epithelium for a period of 1 h, treating with gentamicin for 30 minutes to kill extracellular bacteria, and determining the number of viable intracellular bacteria. We find that InlB is important for efficient invasion since intracellular Δ*inlB* are significantly reduced compared to WT (∼35%, p<0.0001, Figure 2A). At various time points during the 1 h infection, polarized MDCK monolayers were fixed and analyzed by confocal immunofluorescence microscopy with an inside-outside staining protocol that distinguishes attached extracellular bacteria from internalized bacteria. Both WT and Δ*inlB* invade polarized MDCK monolayers almost exclusively through MCJs, which represent only ∼2% of all available junctions (Figure 2B, Figure S2 and [30]), however invasion by Δ*inlB* is delayed. By 20 minutes after adhesion, internalized WT bacteria are observed, while all Δ*inlB* remain extracellular. At each time point after attachment a greater proportion of WT than Δ*inlB* are internalized (Figure 2B, 2C). Thus, InlB is dispensable for cell attachment in polarized epithelia but increases invasion once bacteria are associated with the cell surface.

We also investigated the role of InlB in intracellular replication to determine whether the increase in internalized bacteria is solely due to an accelerated entry of WT bacteria or also due to increased replication within the cell. Polarized MDCK monolayers were infected with WT or Δ*inlB* at a multiplicity of infection (MOI) of 10 bacteria/cell. At various time points, the monolayers were fixed and analyzed by confocal immunofluorescence microscopy to quantify the replication rate of the intracellular bacteria (Figure S2). At each time point during infection WT plaques are greater in area and bacterial number than Δ*inlB* (Figure S2, Figure 2D). However, intracellular doubling times are essentially identical between the two strains (WT T_d_ = 1.25 h and Δ*inlB* T_d_ = 1.26 h; comparison of fits (k), p = 0.97; Figure 2D). Thus, InlB influences the rate of epithelial invasion at MCJs but is not involved in intracellular growth.

### InlB Accelerates Invasion of Polarized MDCK Cells by Activating c-MET from the Apical Compartment

Soluble InlB activates c-Met signaling when added to nonconfluent epithelia with exposed basolateral surfaces [10], [11], [19]. However, since c-Met is a basolateral protein not exposed on the apical side it is unclear whether the same occurs in polarized epithelia [31], [66]. To test the role of c-Met on apical invasion of polarized epithelial cells, we pretreated the confluent polarized monolayers with SU11274 to inhibit c-Met signaling or DMSO as a control, then infected them with WT or Δ*inlB* through the apical compartment [67]. The kinase inhibitor reduces WT invasion to the level of Δ*inlB* invasion but has no significant effect on the invasion of Δ*inlB* (Figure 2E). Thus, c-Met activation is required for InlB activity during apical invasion of the MCJs.

Since c-Met is not readily available in the apical surface, we wondered whether InlB acts as a soluble factor or whether c-Met is activated locally at the MCJs after bacterial attachment. It has been suggested that InlB may function as a soluble and diffuse c-Met agonist since InlB is only loosely associated with the bacterial surface, and since recombinant InlB can mimic HGF by inducing cell membrane ruffling or cell scattering [10], [19], [52], [62], [68]. On the other hand, *Lm* invade cells through tight membrane invaginations without apparent changes of cell surfaces where bacteria are absent, suggesting that InlB associated with the bacterial surface mediates c-Met activation within close proximity to each individual bacterium [14], [63], [69]. We performed co-infections of polarized MDCK monolayers with a mixture of WT and Δ*inlB* and hypothesized that WT would rescue the defect of Δ*inlB* invasion if InlB acts as a soluble factor acting on all cells within the epithelium. We find that InlB does not act globally on the epithelium, since Δ*inlB* continue to exhibit a defect in invasion in the presence of WT in a mixed infection. The magnitude of the defect is the same in mixed as in separate infections (Figure 2A, 2F) and we obtained the same competition defect for Δ*inlB* at MOI ratios of 100∶1, 10∶1, or 1∶1 (Figure 2F, Figure S3).

To further address whether c-Met activation is restricted to the immediate surrounding of individual bacteria, we tested whether the c-Met kinase inhibitor used in a mixed infection would reduce both WT and Δ*inlB* invasion, or alternately whether c-Met inhibition would selectively affect WT invasion. As in single infections, the c-Met kinase inhibitor reduced WT invasion to the level of Δ*inlB* in a mixed infection (Figure 2F, Figure S3). These results indicate that local c-Met activation by InlB at the MCJ is responsible for the increased invasion.

### InlB and HGF Accelerate Endocytosis at Multicellular Junctions

Since InlB increases the rate of *Lm* internalization through activation of c-Met at MCJs, we also wondered whether MCJs are intrinsically different in their endocytic activity as compared to the rest of the apical surface. MCJs represent sites of recent or ongoing cell extrusion where the tight junctions (TJs) are being rapidly remodeled [30], [35]. Additionally, we find that E-cadherin is remodeled through endocytosis during cell extrusion and MCJ formation (Figure S4A). Thus, we hypothesized that MCJs may be more permissive to bacterial entry than other junctional sites because of greater endocytic potential. This is also suggested by the observation that *Lm* invasion through MCJs is more likely than invasion through other junctional sites of attachment: 26% of *Lm* associated with a polarized MDCK epithelium attach to epithelial junctions that are not a MCJ but invasion occurs almost exclusively at MCJs since 97% of intracellular foci of *Lm* originate at these sites, even in the absence of InlB (Figure 2B, Figure S2 and [30]).

**Figure 3:**
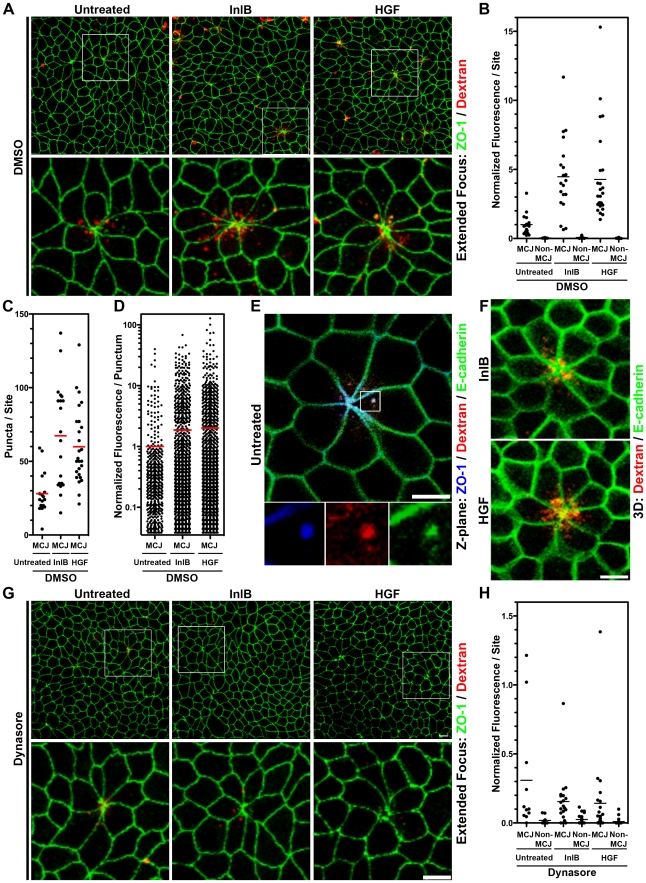
InlB and HGF accelerate dynamin-dependent endocytosis at multicellular junctions. (A) Extended focus views of polarized MDCK monolayers treated with DMSO 30 minutes prior to and including apical treatment with InlB or HGF for 1 h, then apical treatment with dextran, a fluid phase internalization marker, red, for 30 minutes. Monolayers were counterstained for ZO-1, green. (B) Quantification of dextran fluorescence in 50 µm×50 µm regions centered at multicellular junctions (MCJ) or at regions without multicellular junctions (Non-MCJ) of DMSO treated monolayers. (C) Quantification of dextran puncta in 50 µm×50 µm regions centered at MCJs in DMSO treated monolayers. (D) Quantification of dextran fluorescence in all puncta analyzed at MCJs. (E) Single confocal Z-plane through polarized E-cadherin-GFP MDCK cells treated apically with dextran, red, for 30 minutes and counterstained for ZO-1, blue. (F) 3D rendered confocal images E-cadherin-GFP MDCK cells treated apically with InlB or HGF and with dextran, red, for 30 minutes. (G) Extended focus views of polarized MDCK cells treated with dynasore, to inhibit dynamin function, 30 minutes prior to and including apical treatment with InlB or HGF for 1 h, then apical treatment with dextran, red, for 30 minutes. Monlayers were counterstained for ZO-1, green. (H) Quantification of dextran fluorescence in 50 µm×50 µm regions centered at multicellular junctions (MCJ) or at regions without multicellular junctions (Non-MCJ) of dynasore treated monolayers; data normalized to B and displayed with a different scale. Scale bars 10 µm.

To test whether endocytosis is naturally increased at MCJs, we added fluorescent dextran to the apical side of uninfected polarized MDCK monolayers for 30 minutes and determined whether uptake is greater at MCJs than through the rest of the apical surface (Figure 3). Puncta of internalized dextran are readily found in the cells making MCJs and the fluorescence intensity of dextran is higher than at non-multicellular junction (Non-MCJ) regions of the polarized monolayer (Figure 3A, 3E). Interestingly, some internalized dextran at MCJs colocalizes with internalized E-cadherin as well as ZO-1, a scaffolding protein associated with the TJs in polarized cells (Figure 3E) [70]. We observe similar puncta of endocytosed E-cadherin at MCJs *in vivo* at villus tips (Figure S4B). Thus, significant endocytosis occurs specifically at MCJ sites in a polarized epithelium. In addition, E-cadherin, the receptor for *Lm* internalization, is naturally endocytosed at MCJs.

We asked whether c-Met activation at MCJs could locally accelerate endocytosis since growth factor activation of RTKs has been shown to induce endocytosis of E-cadherin through either macropinocytosis or clathrin-mediated endocytosis [46], [71], [72]. We pretreated polarized MDCK cells from the apical side for 1 h with HGF or InlB prior to the addition of fluorescent dextran to the apical compartment. We find that both HGF and InlB significantly increase the amount of dextran endocytosed at MCJs (p<0.001), but not at non-MCJ regions compared to untreated cells (Figure 3A–B, Figure S5). To control for the specificity of this process we used a truncated InlB consisting of only the C-terminal GW domains (GW[2]–[3]) and this has no effect on endocytosis compared to untreated monolayers (Figure S5) [15]. These results suggest that basolateral c-Met is made transiently accessible through the rapid junctional remodeling at MCJs. Puncta of endocytosed dextran were also co-localized with junctional proteins at MCJs in HGF and InlB treated monolayers (Figure 3A, 3F). Increased endocytosis of dextran after HGF and InlB treatment is the product of an increase in the number of puncta of internalized dextran per MCJ (Untreated versus InlB or HGF p<0.001, Figure 3C) and an increase in the amount of dextran internalized as determined by fluorescence intensity per punctum (Untreated versus InlB p<0.05, Untreated versus HGF p<0.01, Figure 3D). This suggests that both the rate of endocytosis as well as the capacity of individual endocytic vesicles is increased by HGF or InlB.

### Endocytosis and *L. monocytogenes* Invasion at Multicellular Junctions Require Common Endocytic Machinery

**Figure 4:**
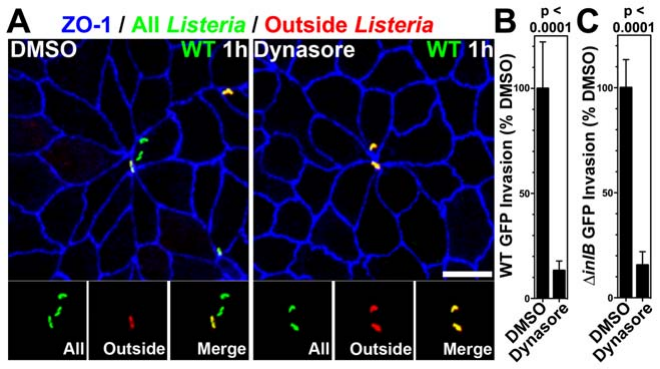
Dynamin-dependent invasion of MCJs. (A) Polarized MDCK cells were pretreated with DMSO or dynasore for 30 minutes and then infected with GFP-expressing WT *Lm* for 1 h. To evaluate intracellular versus extracellular bacteria, we performed an outside staining where extracellular adherent GFP-expressing *Lm* were stained before permeabilization in red. Intracellular *Lm* are green and external *Lm* are a combination of red/green or yellow. Monolayers were counterstained with antibodies to ZO-1, blue. Scale bars 10 µm. (B) Quantification of relative WT *Lm* invasion of Polarized MDCK monolayers pretreated with DMSO or dynasore for 1 h. Relative mean and SD of intracellular CFUs recovered after gentamicin treatment are shown. p<0.0001. (C) Quantification of relative Δ*inlB Lm* invasion of Polarized MDCK monolayers treated with DMSO or dynasore. Relative mean and SD of intracellular CFUs recovered after gentamicin treatment are shown. p<0.0001.

In nonpolarized cells, *Lm* invasion requires molecular machinery associated with clathrin-mediated endocytosis, including dynamin [59], [73]. To test whether invasion of the MCJs is also dynamin-dependent, we pretreated polarized MDCK monolayers with either DMSO as a control or dynasore, an inhibitor of dynamin, and infected them with *Lm* (Figure 4) [74]. Using inside-outside confocal microscopy analysis of monolayers infected for 1 h, we find that *Lm* invade control cells at MCJs, but cannot invade cells treated with dynasore (Figure 4A). A second assay using gentamicin protection also confirmed this result. *Lm* were allowed to invade for a period of 1 h, the infected monolayers were treated with gentamicin for 30 minutes and the number of viable intracellular bacteria was determined. Compared to control cells, polarized cells treated with dynasore are significantly less permissive for *Lm* invasion (Figure 4B–C; ∼13% DMSO, WT p<0.0001; ∼16% DMSO, Δ*inlB* GFP p<0.0001)

To test whether the increased rate of apical endocytosis at MCJs is also a dynamin-dependent process, we pretreated polarized MDCK monolayers with dynasore or DMSO as a control prior to addition of fluorescent dextran. Pretreatment of polarized cells with dynasore inhibits nearly all endocytosis of dextran at multicellular junctions regardless of HGF or InlB treatment (Figure 3F, 3G). Indeed, uptake at multicellular junctions is not significantly higher than uptake at non-multicellular junctions within monolayers treated with dynasore (Figure 3G). These data suggest that InlB accelerates dynamin-dependent endocytosis at MCJs leading to an increase the rate of *Lm* uptake at these sites.

### InlB Enhances Apical Endocytosis at the Villus Tip Extrusion Zone

**Figure 5:**
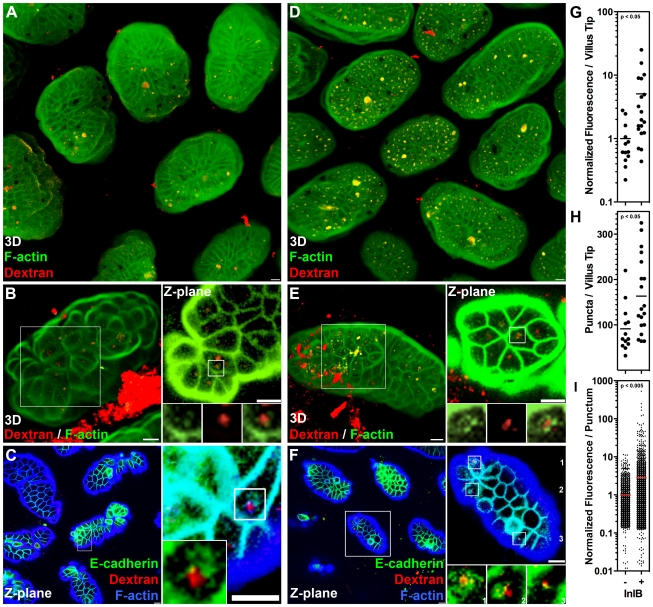
InlB enhances endocytosis at the intestinal villus tips. Fluorescent dextran, a fluid phase internalization marker, with or without InlB was incubated for 45 minutes in mouse ileal loops. (A) 3D confocal reconstructions of villus tips from an ileal loop incubated with dextran, red. Tissue was counterstained with phalloidin for F-actin, green. (B) Higher magnification villus tip from an ileal loop incubated with dextran, and a single Z-plane from inset showing puncta of intracellular dextran. (C) Single Z-plane from confocal image of villus tips from a loop incubated with dextran, red, and counterstained with anti-E-cadherin antibodies, green, and with phalloidin for F-actin, blue. Inset shows higher magnification of dextran puncta and intracellular E-cadherin. (D) 3D confocal reconstructions of villus tips from an ileal loop incubated with dextran, red, and InlB. Tissue was counterstained with phalloidin for F-actin, green. (E) Higher magnification villus tip from an ileal loop incubated with dextran, red, and InlB, and a single Z-plane from inset showing puncta of intracellular dextran. (F) Single Z-plane from confocal image of villus tips from a loop incubated with dextran, red, and InlB and counterstained with anti-E-cadherin antibodies, green, and phalloidin for F-actin, blue. Inset shows higher magnification of three separate dextran puncta and intracellular E-cadherin. Scale bars 10 µm. (G) Quantification of dextran fluorescence per villus tip from ileal loops incubated with dextran only (−) or dextran and InlB (+). (H) Quantification of dextran puncta per villus tip from ileal loops incubated with dextran only (−) or dextran and InlB (+). (I) Quantification of dextran fluorescence from all puncta analyzed from villus tips of ileal loops incubated with dextran only (−) or dextran and InlB (+).

Our tissue culture results suggested that the villus tip extrusion zone might also be permissive to *Lm* invasion because of an increased rate of endocytosis *in vivo*. We incubated fluorescent dextran in mouse ileal loops for 45 minutes and examined villus tips by confocal microscopy to test this hypothesis (Figure 5). Puncta of fluorescent dextran are readily found in the villus tip epithelium, but not the epithelium along the lateral sides of villi or crypt epithelium (Figure 5A, 5B and data not shown). To test whether InlB promotes endocytosis at the villus tips, we incubated InlB with dextran in mouse ileal loops (Figure 5D, 5E). Internalized puncta of dextran at MCJs are found associated with E-cadherin in both untreated and InlB treated villi (Figure 5C, 5F). However, InlB significantly increases the amount of dextran endocytosis at the villus tips (p<0.05, Figure 5G) by increasing the number of puncta of dextran per villus tip (p<0.05, Figure 5H) and the amount of dextran per punctum (p<0.005, Figure 5I).

## Discussion

Epithelia are the first site of interaction between the host and a wide variety of invading pathogens and the intercellular junctions are crucial to maintain a tight seal between epithelial cells to prevent microbial invasion. It is interesting that diverse microbes have evolved strategies to usurp the epithelial junctions to mediate extracellular colonization, intracellular invasion or paracellular breach (reviewed in [75]–[79]).

Microbes that invade epithelial cells often use receptors for internalization that are part of the junctions or are basolateral proteins. For example, reoviruses bind JAM-A, coxsackie and adenovirus bind CAR, hepatitis C virus binds claudins and occludin, rotaviruses, *Shigella flexneri* and enteropathogenic *Yersiniae* bind integrins, α-herpesviruses bind Nectins, and *Listeria monocytogenes* (*Lm*) binds E-cadherin [7], [80]–[97]. Although targeting of junction or basolateral proteins by invasive pathogens is a successful strategy, it is also seemingly paradoxical since these receptors are not normally localized at the apical surface.

**Figure 6:**
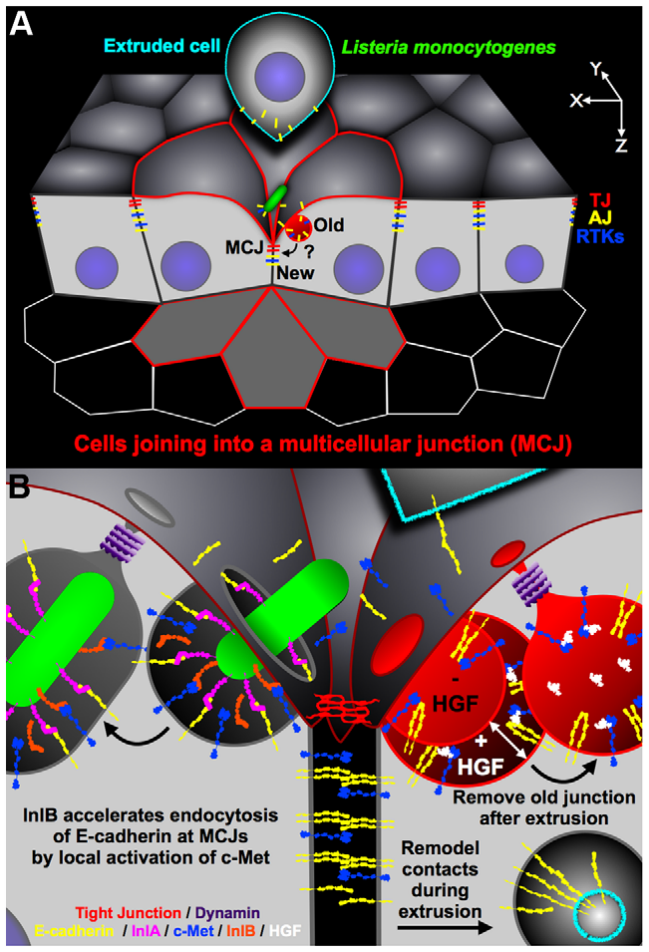
Diagram of *Lm* invasion coupled to junctional remodeling by endocytosis at the MCJ. (A) *Lm* find exposed E-cadherin at multicellular junctions (MCJs) when extruded cells are displaced from the epithelium. New tight junctions (TJs) and adherens junctions (AJs) are formed below the extruded cell at the invagination of MCJs. The old junctions and intercellular-adhesions are removed by endocytosis. RTKs, receptor tyrosine kinases. (B) Close up diagram of the region of bacterial invasion at the MCJ. Left: after binding to E-cadherin via InlA, InlB activates c-Met which is also abnormally exposed to the apical surface at MCJs. Activation of c-Met locally by InlB at the MCJ increases the rate of endocytosis of *Lm*. Right: endocytic removal of the old junctions parallels *Lm* uptake. Experimentally, c-Met activation by HGF may increase the rate of formation and the size of endosomes. E-cadherin, yellow; InlA, magenta; c-Met, blue; InlB, orange; HGF, white; dynamin, purple; a simplified tight junction, red.

The study of *Listeria* pathogenesis in the gastrointestinal tract reveals that *Lm* has evolved to target a subset of intercellular junctions that have a natural and transient defect in cell polarity generated during the process of cell extrusion. First, we noted that *Lm* uses InlA to access E-cadherin as it becomes exposed at multicellular junctions (MCJs, Figure 6A) [30]. Our studies here of InlB suggest that the MCJ's are not only a natural site of local loss of polarity, but also that the normal process of junction renewal involves accelerated endocytic processes that can be hijacked and modulated by additional bacterial invasive factors (Figure 6B).

Why are MCJs inherently endocytic? The formation and resolution of MCJs by cell extrusion requires junctional reorganization, changes in cell position, and changes in cell morphology [30], [35], [38], [98]. There is increasing evidence that remodeling of adhesive contacts, including modification of junctional length or cell position within epithelia, requires endocytosis of adhesion molecules such as E-cadherin [99]–[104]. Furthermore, it was found that in cells neighboring extruding cells, large endosome-like structures contain tight junction (TJ) strands [35]. We also find that cells neighboring extruding cells internalize E-cadherin, a component of the adherens junction (AJ), from the extruding cell while forming a MCJ (Figure S4, Figure 5B). Thus endocytosis at MCJs may be important to release adhesive contacts between the extruding cell and the rest of the epithelium, for removal of lumenally exposed basolateral and junctional proteins, and for redistribution of cell shape and position during cell extrusion [35], [99]–[101], [103]–[105]. It has been suggested that *Lm* adherence and invasion via E-cadherin is analogous to AJ assembly because of the similarity of their molecular requirements [28], [63], [106], [107]. However, our model suggests that *Lm* invasion subverts junction disassembly, rather than assembly (Figure 6). This concept is supported by the fact that InlA binding results in tyrosine phosphorylation, ubiquitination and endocytosis of E-cadherin [27].

InlA binding to E-cadherin is sufficient for *Listeria* invasion, however modulation of endocytosis by InlB accelerates this process (Figure 6B). We show that while InlB is dispensable for attachment, it synergistically promotes invasion of MCJs through activation of c-Met kinase signaling. Activation of cell signaling that results in endocytosis is a strategy utilized by other invasive microbes. For example, viruses like coxsackievirus, HIV, caposi's sarcoma-associated herpesvirus and adenovirus, and bacteria like *Salmonella, Shigella, Brucella, Neisseria, Mycobacteria, Haemophilus and Legionella* can trigger macropinocytosis or macropinocytosis-like processes [80], [108]–[123]. In contrast, *Lm* utilizes a so-called ‘zipper-like’ mechanism of invasion/endocytosis distinct from macropinocytosis [14], [69]. Other investigators have shown that *Lm* requires dynamin and other molecular components of clathrin-mediated endocytosis for efficient invasion of nonpolarized cells [55], [59], [73]. Furthermore, macropinocytosis is thought to be independent of dynamin and requiring an alternate pinchase [124], [125]. It has been suggested that *Lm* hijacks the actin- and dynamin-dependent internalization of clathrin-coated paques, which are larger than clathrin-coated pits [126], [127]. We also find that *Lm* invasion of a polarized epithelium through the MCJs requires functional dynamin even in the absence of InlB. Similarly, *L. innocua* expressing InlA, but not InlB, requires functional dynamin for invasion [128]. This further supports the notion that *Lm* subverts junction disassembly since both *Lm* invasion via E-cadherin and junction regulation via E-cadherin endocytosis require functional dynamin [102], [129].

InlB has been shown to promote dynamin-dependent internalization of *Listeria* when the bacteria have access to the basolateral surface, and HGF similarly promotes internalization of E-cadherin when added to basal surfaces [59], [71]. Although c-Met is not exposed on the apical surface of epithelia, we hypothesized that InlB could activate c-Met because of the local loss of cell polarity that occurs at MCJs. We find that apical treatment of polarized epithelia with either HGF or InlB increases apical endocytosis of dextran at MCJs (Figure 6B). Interestingly HGF and InlB do not increase endocytosis at non-MCJ regions of the epithelia suggesting that c-Met, a basolateral protein like E-cadherin, is also only accessible from the apical side through the process of cell extrusion and MCJ formation [30]. We confirmed these results *in vivo* showing that purified InlB added from the lumenal side increases endocytosis of fluorescent dextran at the extrusion zone of the intestinal villus tip.

We provide here the first evidence that InlB is involved in intestinal invasion. Other studies have failed to identify a role for InlB in the intestinal phase of infection [20], [24]. However, the contribution of InlB to infection may have been difficult to discern at late time points because most studies utilize severe systemic disease as an endpoint of infection, or because of high variation in animal to animal infections. Additionally, other studies of enteric Listeriosis have used treatments that neutralize stomach acid. This may suppress expression of *inlA and inlB*, which are upregulated by an acid stress response [39]–[42]. In contrast, we did not alter the acid environment and also developed a coinfection assay that allows for precise quantification of *Lm* in the villus tips of the same animal at early time points. Although the effect of InlB for promoting invasion of the villus tips is not large, it is comparable in magnitude of the role of InlB for invasion of cultured epithelial cells. In addition, it is comparable in magnitude to the recently discovered role for InlB in placental invasion after intravenous infection, an experimental route that bypasses the gastrointestinal tract and prior cell invasion [20]. Our study has focused only on the role of InlB in modulating *Lm* invasion of a very specific site, the MCJ. Future investigation will address whether InlB affects the pathophysiology of gastrointestinal colonization and of invasive Listeriosis after oral infection.

In summary, we have explored the mechanisms of *Lm* invasion of polarized epithelia, the first stage of an infection that can range from asymptomatic colonization, to self-limiting enteritis, to potentially deadly invasive and disseminated disease. Our mechanistic model demonstrates how two microbial invasins with different receptors and different adhesin properties can function cooperatively to promote invasion of the intestinal villus tips (Figure 6). The process of cell extrusion requires junctional remodeling and removal of adhesive contacts that allows the dying cell to detach from the epithelium (Figure 6A). After the cell has been extruded, basolateral proteins from the old junction must be removed from above the newly formed TJ on the surrounding cells at the MCJ (Figure 6A, 6B). As an evolutionary strategy, it is interesting that *Lm* targets junction remodeling and dynamin-dependent removal of E-cadherin from the cell surface as a mechanism of internalization rather than binding a more accessible, but more stable, apical receptor. This concept should be relevant to the study of other microbes that target junctional receptors. Without InlB, *Lm* invasion is less efficient. Without InlA, InlB does not provide adhesive strength for *Lm* to bind to the epithelium. Since activation of c-Met results in the co-endocytosis of both receptors, InlB has evolved to provide a local increase in junctional remodeling that allows for enhanced dynamin-dependent *Lm* internalization (Figure 6B).

## Materials and Methods

### Ethics Statement

All animal experiments were performed in accordance to NIH guidelines, the Animal Welfare Act, and US federal law. Such experiments were approved by Stanford University's Administrative Panel on Laboratory Animal Care (A-PLAC), which has been accredited by the Association of Assessment and Accreditation of Laboratory Animal Care International (AAALAC). All animals were housed in a centralized and AAALAC-accredited research animal facility that is fully staffed with trained husbandry, technical, and veterinary personnel.

### Chemicals and Reagents

A stock of 5 µg/ml HGF in H_2_O 0.1% BSA was stored at −80°C until dilution at use (Sigma-Aldrich, St. Louis, MO). InlB-His6 and a truncated variant containing only the terminal GW domains, GW[2]–[3]-His6 at ∼25 mg/ml in 10 mM sodium acetate pH 4.5, 1 mM DTT, 0.5 mM EDTA were purified as described in [15], [16] and stored −80°C until dilution at use. c-Met Inhibitor SU11274 and dynamin inhibitor dynasore ([67], [74]; Calbiochem, San Diego, California) were stored in DMSO at −20°C until dilution at use. A stock of Neutral fixable Texas Red 10 kDa dextran (Molecular Probes, Eugene, Oregon) was stored at 25 mg/ml in DMEM at −20°C until dilution at use.

### Cloning and Generation of *L. monocytogenes* Strains Expressing *inlA^m^*, *inlA* ^m^ *B* and sGFP

The tRNA^ARG^ site-specific shuttle integration vectors pPL3 and pPL3e, which respectively confer chloramphenicol and erythromycin resistance to *Listeria*, and the *L. monocytogenes* (*Lm*) strain DH-L1039, which expresses sGFP under the control of the Hyper-SPO1 promoter fused to the 5′ UTR of *hly* (pHyperSPO1-*hly*5′UTR-sGFP), were the kind gifts of Dr. Darren E. Higgins (Harvard University, Boston, Massachusetts) [130]. pHyperSPO1-*hly*5′UTR-sGFP was PCR amplified from DH-L1039 genomic DNA with primers 37/33 (Table 1). SalI digested pHyperSPO1-*hly*5′UTR-sGFP was ligated with SalI digested pPL3 or pPL3e to generate pMP74 or pMP76, respectively.

**Table 1:** Oligonucleotides Used in This Study.

#	Name	Sequence (5′ to 3′)	Purpose or Reference
1	InlA_Coding_Forward	cgggatccaacgagccaaccgtgg	Anneals ∼700 bp 5′ of *inlA*. Underlined BamHI site. [25]
2	InlA_Coding_Reverse	cgggatcctctccgcttgtactttcgcc	Anneals ∼180 3′ of *inlA*. Underlined BamHI site. [25]
3	InlAB_Coding_Reverse	cgggatccttatttctgtgcccttaaattagc	Anneals at transcriptional stop site of *inlB*. Underlined BamHI site. [25]
27	NC16	gtcaaaacatacgctcttatc	Anneals 5′ to tRNA^ARG^ in *L. monocytogenes* genome. [131]
28	PL95	acataatcagtccaaagtagatgc	Anneals within PSAint in pPL2, pPL3 and pPL3e. Used with NC16 to verify pPL2, pPL3 and pPL3e plasmid integration. [131]
33	salI_gfpmut2_reverse	aggtcgacttatttgtatagttc	Anneals at transcriptional stop site of GFP. Used to amplify sGFP expression construct from DH-L1039. Underlined SalI site. [130]
37	#2_salI_pHYSPO1	ccgtcgacaattttgcaaaaagttgttgacttt	Anneals at 5′ of Hyper SPO1 promoter. Used to amplify sGFP expression construct from DH-L1039. Underlined SalI site. [130]
47	InlA_S192N	gctttcaggtttaactaatctacagcaattaaattttggtaatcaagtgacaga	Quickchange mutagenesis of *inlA*
48	InlA_S192N_antisense	tctgtcacttgattaccaaaatttaattgctgtagattagttaaacctgaaagc	Quickchange mutagenesis of *inlA*
49	InlA_Y369S	gtttaacaaagcttcaaagattatttttcagtaataacaaggtaagtgacgtaagctcac	Quickchange mutagenesis of *inlA*
50	InlA_Y369S_antisense	gtgagcttacgtcacttaccttgttattactgaaaaataatctttgaagctttgttaaac	Quickchange mutagenesis of *inlA*

*inlA* and *inlAB* were PCR amplified from WT *Lm* 10403S genomic DNA with primers 1/2 and 1/3, respectively, as in [25]. *inlA* or *inlAB* were ligated with pCR4-BluntTOPO (Qiagen, Valencia, CA) and subjected to two rounds of Quickchange site-directed mutagenesis (Stratagene, La Jolla, CA) with primer pairs 47/48 and 49/50 to introduce S192N and Y369S mutations into *inlA* and generate the murinized variants *inlA^m^* or *inlA^m^B* (Table 1). *inlA^m^* or *inlA^m^B* were digested with BamHI and ligated with BamHI digested pPL3, pPL3e, pMP74 or pMP76. These constructs were transformed into SM10 (λpir), and introduced to *Lm* by conjugative mating as described in [131] (Table 2). Integration was confirmed with primers NC16/PL95 as described in [131] (Table 1).

**Table 2:** *L. monocytogenes* Strains Used in This Study.

Strain	Relevant Characteristics*	In text/figures as:
10403S	Wild type (WT) *L. monocytogenes* serotype 1/2a.	-
DP-L4406	10403S Δ*inlB* [25]	-
DP-L4404	10403S Δ*inlAB* [25]	-
DH-L1039	WT sGFP, cm^R^ [130]	-
LM 124	WT sGFP, cm^R^ (this study)	WT (cm^R^)
LM 126	Δ*inlB* sGFP, cm^R^ (this study)	Δ*inlB* (cm^R^)
LM 128	WT sGFP, em^R^ (this study)	WT
LM 130	Δ*inlB* sGFP, em^R^ (this study)	Δ*inlB*
LM 101	Δ*inlAB inlA* ^m^, sGFP, cm^R^ (this study)	Δ*inlB* ^m^ GFP (cm^R^)
LM 102	Δ*inlAB inlA* ^m^, sGFP, em^R^ (this study)	Δ*inlB* ^m^ GFP
LM 106	Δ*inlAB inlA* ^m^ *B*, sGFP, cm^R^ (this study)	WT^m^ GFP (cm^R^)
LM 111	Δ*inlAB inlA* ^m^ *B*, sGFP, em^R^ (this study)	WT^m^ GFP
LM 159	Δ*inlAB inlA* ^m^, em^R^ (this study)	Δ*inlB* ^m^
LM 163	Δ*inlAB inlA* ^m^ *B*, em^R^ (this study)	WT^m^

*GFP, green fluorescent protein; cm^R^, chloramphenicol resistant at 7.5 µg/ml; em^R^, erythromycin resistant at 5 µg/ml.

### Bacterial Strains and Culture Conditions

*Lm* strains are listed in Table 2. *Lm* were grown on BHI agar or in BHI broth (BD/Difco, San Jose, California) supplemented with streptomycin at 200 µg/ml, chloramphenicol at 7.5 µg/ml or erythromycin at 5 µg/ml, when appropriate. One-shot Top10 *E. coli* (Invitrogen, Carlsbad, California), used for general cloning steps, was cultured in LB broth and on LB agar supplemented with kanamycin at 50 µg/ml or choramphenicol at 25 µg/ml, when appropriate. *E. coli* strain SM10 (λpir) was kindly provided by Dr. Denise Monack (Stanford University, Stanford, California). *E. coli* SM10 (λ pir), as the donor for bacterial conjugation, was cultured in LB supplemented with kanamycin at 30 µg/ml and chloramphenicol at 25 µg/ml, when appropriate.

### Cell Culture and Infection

MDCK II, MDCK II E-cadherin-GFP and MDCK II E-cadherin-RFP cells were kindly provided by W. James Nelson (Stanford University, Stanford, California) [132], [133]. Cells were maintained at 37°C in 5% CO_2_ atmosphere in DMEM (Gibco, San Diego, California) supplemented with 5% fetal bovine serum (FBS, Gibco). For infection experiments, cells were trypsinized and seeded on 12 well polycarbonate tissue culture dishes or 12 mm polycarbonate tissue culture inserts (Transwell filters; Costar, Cambridge, Massachusetts) at a density of 10^6^ cells/cm^2^ and supplemented with fresh media daily for 4 days. For experiments with inhibitors, DMEM 2.5 µM c-Met Inhibitor SU11274/0.15% DMSO was added to the monolayers 12 h prior to infection or DMEM 80 µM dynasore/0.1% DMSO was added to the monolayers 30 min or 1 h prior to infection. *Lm* infections (multiplicity of infections, MOIs, of 1∶1 to 100∶1) and assays of attachment invasion were performed essentially as described in [30]. To assay for intracellular replication, polarized MDCK monolayers were infected with an MOI of 10 bacteria/cell for a 10 minutes to allow attachment and were then washed 4X with DMEM to remove unadhered bacteria. Six to ten plaques per time point were randomly found and imaged by 3D confocal microscopy without regard to size or bacterial number and subsequently analyzed for bacterial number from all acquired images. Prism software (GraphPad, San Diego, California) was utilized for construction of graphs and for statistical analysis of data. Student's t-test was used to compare two sample groups. ANOVA with Bonferroni's post-tests was used to analyze 3 or more sample groups. The competitive index (C.I.) of two strains was determined as C.I. =  (Stain A output/Strain B output)/(Stain A input/Strain B input).

### *Listeria* Infection of Mice

*Lm* cultures were grown at 30°C overnight in BHI without agitation, pelleted and resuspended in phosphate buffered saline (PBS). Female 8-week old BALB/c mice (obtained at 6–7 weeks from The Jackson Laboratory, Bar Harbor, Maine) were food restricted overnight but allowed free access to water and inoculated with a feeding needle intragrastrically with a maximum volume of 200 µl. Mice were then immediately allowed free access to food and water.

### Dextran Endocytosis in Tissue Culture

MDCK II or MDCK II E-cadherin-GFP cells were trypsinized and seeded on 12 mm polycarbonate Transwell tissue culture inserts at a density of 10^6^ cells/cm^2^ and supplemented with fresh basal media daily for 5 days. The media was changed to plain DMEM 80 µM Dynasore/0.1% DMSO or DMEM 0.1% DMSO at −1:30 hours. A final concentration of 1 µg/ml InlB or GW[2]–[3], or 0.1 µg/ml HGF was added at −1:00 h to the apical side and at time 0:00 1 mg/ml neutral fixable Texas Red 10 kDa dextran was added to the apical side for 30 minutes. Monolayers were washed 4X to remove extracellular dextran and monolayers were fixed and processed for immunofluoresence microscopy, as described in [30]. Confocal images were analyzed using Volocity software (Improvision, Lexington, Massachusetts). To quantify and quantitatively describe intracellular fluorescent dextran puncta, an analysis script was designed to find objects within 5–100% fluorescence intensity, exclude objects less than 0.5 µm^3^ or greater than 100 µm^3^ and separate touching objects with an object size guide of 0.1 µm^3^. The data were clipped to a square region of interest 50 µm×50 µm centered at a multicellular junction (MCJ) or at non-MCJ regions.

### Dextran Endocytosis in Mouse Ileal Loops

BALB/c mice (The Jackson Laboratory) were fasted overnight prior to surgery but allowed free access to water. Anesthesia was induced by intraperitoneal injection with a mixture of ketamine (40 mg/kg) and xylazine (4–5 mg/kg) in water and the animal was kept on a 37°C pad for the duration of the procedure. For each mouse a midline laparotomy was performed to expose the bowel. The ileocecal junction was identified, and the ileum was ligated with a silk tie just proximal to the cecum. A second circumferential ligature was placed ∼4 cm proximal. A suspension of 2.5 mg/ml neutral fixable Texas Red 10 kDa dextran with or without 10 µg/ml InlB in dPBS was inoculated via a hypodermic needle into the loop (∼50 µl/cm). The intestine was returned to the abdominal cavity and the incision was closed with surgical staples. The mouse was kept under anesthetic for 45 minutes at which time the animal was euthanized and intestines were removed and fixed for whole-mount confocal microscopy imaging, as described in [30]. Confocal images were analyzed using Volocity software (Improvision). To quantify and quantitatively describe intracellular fluorescent dextran puncta, an analysis script was designed to find objects within 5–100% fluorescence intensity, exclude objects less than 1 µm^3^ or greater than 20 µm^3^. The data were clipped to region of interest surrounding each villus tip analyzed.

### Microscopy and Antibodies

Live-cell time-lapse microscopy was performed essentially as described in [134]. Confocal immunofluorescence microscopy was performed as described in [30]. *Lm* were detected by incubation of samples with biotin-conjugated rabbit anti-*L. monocytogenes*, all antigens (YVS4207, Accurate Chemical & Scientific Corp., Westbury, NY; 1∶100 for tissue, 1∶600 for tissue culture). Tight junctions were detected by incubating samples with mouse anti-ZO-1 antibodies (Zymed, South San Francisco, California; 1∶300 dilution). E-cadherin was detected with mAb anti-E-cadherin (BD Transduction Labs, San Jose, California; 1∶600 dilution). Alexa-fluor conjugated streptavidin or Anti-IgG Alexa-fluor conjugated antibodies of appropriate species reactivity and fluorescence spectra were used for secondary detection (Molecular Probes). An immunofluorescence inside/outside staining that distinguishes extracellular from intracellular *L. monocytogenes* was modified from [135] with appropriate antibodies for this study. All nuclei were visualized by incubating samples with TOPRO-3 (Molecular Probes). F-actin was visualized by incubating samples with Alexa-fluor conjugated phalloidins (Molecular Probes).

## Supporting Information

Figure S1 InlB-mediated Colonization of Intestinal Villus Tips From Single Infections of Mice. Mice were infected with 10^10^ CFU WT^m^ GFP (cm^R^) or Δ*inlB* ^m^ GFP (cm^R^) for 5 h. (A–B) Expanded Figures 1B–C showing additional Z-planes with intracellular *Listeria*. Scale bars 10 µm. (C) Villus tips in the terminal ileum were analyzed by microscopy as in A–B and *Listeria* per infected villus tip was quantified. N, the number of infected villi found in the ∼1 cm^2^ whole mount tissue sections analyzed.
(4.49 MB TIF)

Figure S2 WT and Δ*inlB* Intracellular Plaque Formation. Representative confocal immunofluorescence micrographs used to generate data in Figure 2D. Polarized MDCK monolayers were infected with WT or Δ*inlB Lm*, green, fixed at the indicated time points post infection, and stained with phalloidin for F-actin, red. Top panels represent a central X-Y-Z plane and lower panels are extended focus views of the same showing all *Lm*. Scale bars 10 µm.
(8.74 MB TIF)

Figure S3 Expanded Figure 2F, InlB Promotes Invasion Local to the Bacterium. To determine whether the invasion defect of Δ*inlB* could be rescued, confluent MDCK monolayers were either untreated or treated with c-Met inhibitor prior and during infection with a 1∶1 ratio of WT∶WT, as a control, or WT∶Δ*inlB* at an MOI of 100∶1, 10∶1, or 1∶1 bacteria/cell. The ratio of the strains recovered, C.I., after gentamicin treatment was determined.
(0.18 MB TIF)

Figure S4 Endocytosis of E-cadherin at MCJs. (A) E-cadherin endocytosis during cell extrusion and MCJ formation. MDCK and MDCK E-cadherin-RFP cells were mixed and co-cultured to form a confluent monolayer for 1 day and then observed by DIC and fluorescence time-lapse microscopy. An extruding MDCK E-cadherin-RFP cell is marked with an asterisk and neighboring MDCK cells are numbered. Time in minutes, m, is indicated. Arrows indicate puncta of E-cadherin-RFP internalized by non-fluorescent neighboring cells during MCJ formation. (B) E-cadherin remodeling at an MCJ at the villus tip extrusion zone. Mouse intestinal tissue was stained with antibodies to E-cadherin, red, and with Topro-3 to visualize nuclei, blue, and imaged by 3D confocal microscopy. Depth from the apical cell surface, Z, is indicated. Arrows indicate intracellular puncta of E-cadherin. Scale bar 10 µm.
(10.10 MB TIF)

Figure S5 InlB and HGF, but not GW[2]–[3] Accelerate Endocytosis at MCJs. E-cadherin-GFP expressing MDCK monolayers were polarized on Transwell filters for 5 days and then treated with InlB, a truncated InlB containing only the C-terminal GW domains (GW[2]–[3]) or HGF for 1 h and then additionally treated with dextran, a fluid phase internalization marker, red, for 30 minutes. (A) 3D rendered views of polarized E-cadherin-GFP MDCK monolayers. Insets show multicellular junctions (MCJs). Scale bars 10 µm. (B) Quantification of dextran fluorescence in 50 µm×50 µm regions centered at multicellular junctions. (C) Quantification of dextran puncta in 50 µm×50 µm regions centered at MCJs. (D) Quantification of dextran fluorescence in all puncta analyzed at MCJs.
(0.99 MB TIF)

Video S1 Mouse Villus Tip Infected with WT^m^ GFP *L. monocytogenes* 6 h Post Enteric Infection. QuickTime movie of a complete optical scan through a mouse intestinal villus tip infected with WT^m^ *L. monocytogenes*, expressing GFP, green, and counterstained with phalloidin, red, to visualize the F-actin cytoskeleton, and with Topro-3 to visualize nuclei. The movie shows a 3D-rendered villus with multicellular junctions at the tip and then scans the tip through optical sections to show intracellular *Lm*. The tip is then rendered again in 3D to show intracellular bacteria with actin comet tails. Finally, the villus is rotated to see the infecting bacteria from within the epithelium.
(9.79 MB MOV)
