# Supplementary figures and images for: Listeria monocytogenes Internalin B Activates Junctional Endocytosis to Accelerate Intestinal Invasion
Source: PLoS Pathog. 2010 May 13;6(5):e1000900. doi: 10.1371/journal.ppat.1000900 (PMC2869327; doi:10.1371/journal.ppat.1000900)

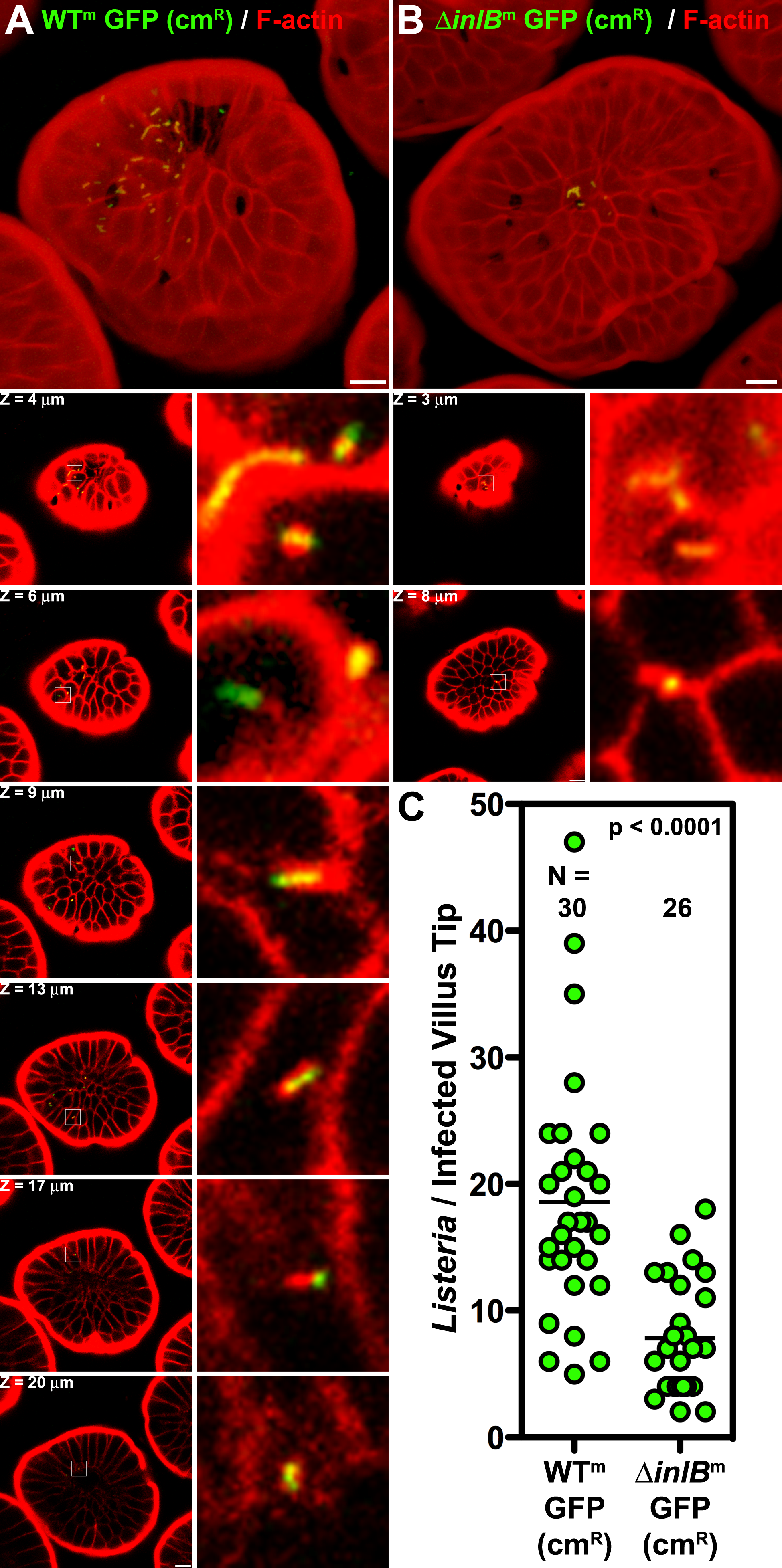

Supplement: Figure S1 — InlB-mediated Colonization of Intestinal Villus Tips From Single Infections of Mice. Mice were infected with 1010 CFU WTm GFP (cmR) or ΔinlB m GFP (cmR) for 5 h. (A–B) Expanded Figures 1B–C showing additional Z-planes with intracellular Listeria. Scale bars 10 µm. (C) Villus tips in the terminal ileum were analyzed by microscopy as in A–B and Listeria per infected villus tip was quantified. N, the number of infected villi found in the ∼1 cm2 whole mount tissue sections analyzed. (4.49 MB TIF) [file ppat.1000900.s001.tif]

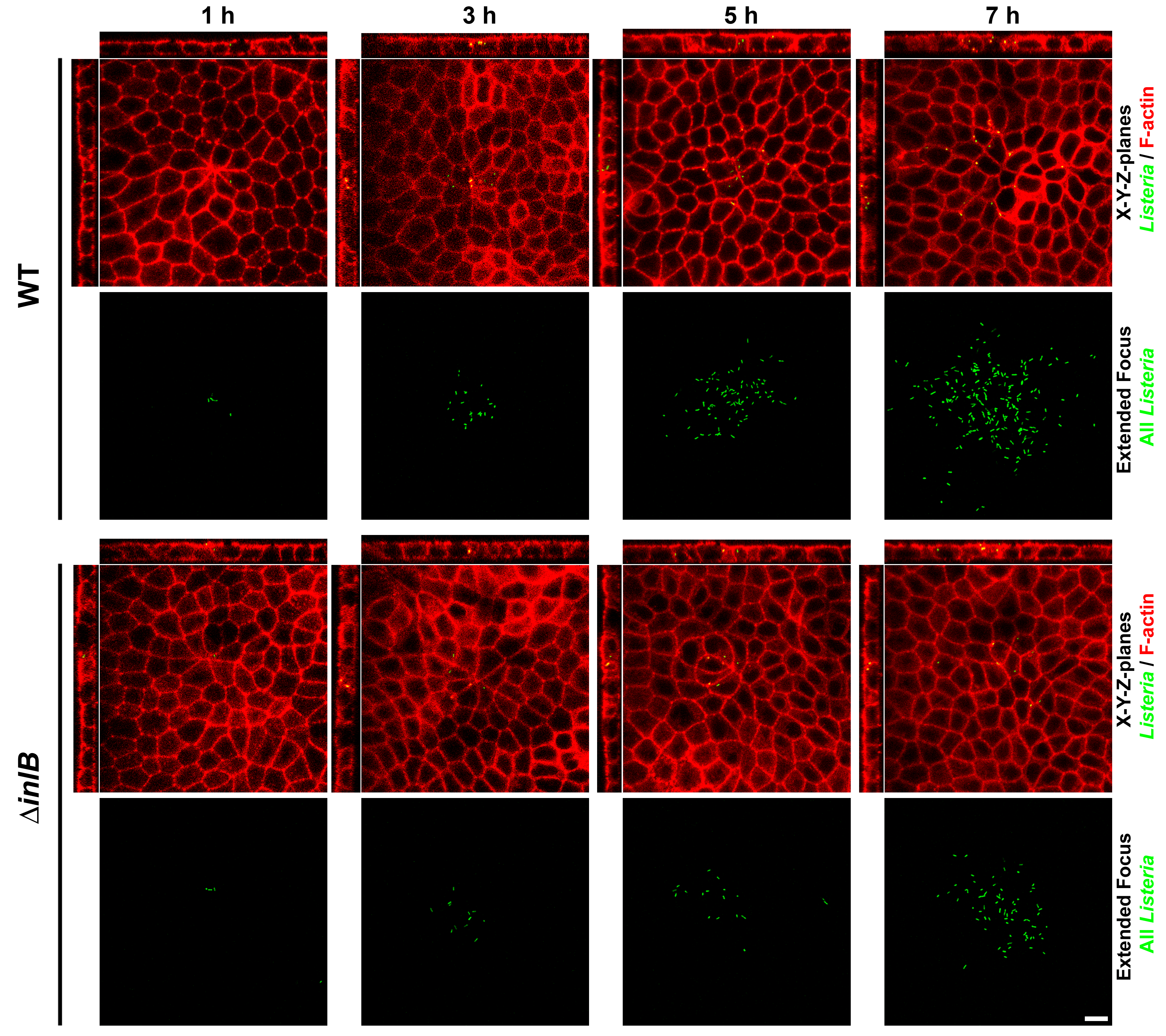

Supplement: Figure S2 — WT and ΔinlB Intracellular Plaque Formation. Representative confocal immunofluorescence micrographs used to generate data in Figure 2D. Polarized MDCK monolayers were infected with WT or ΔinlB Lm, green, fixed at the indicated time points post infection, and stained with phalloidin for F-actin, red. Top panels represent a central X-Y-Z plane and lower panels are extended focus views of the same showing all Lm. Scale bars 10 µm. (8.74 MB TIF) [file ppat.1000900.s002.tif]

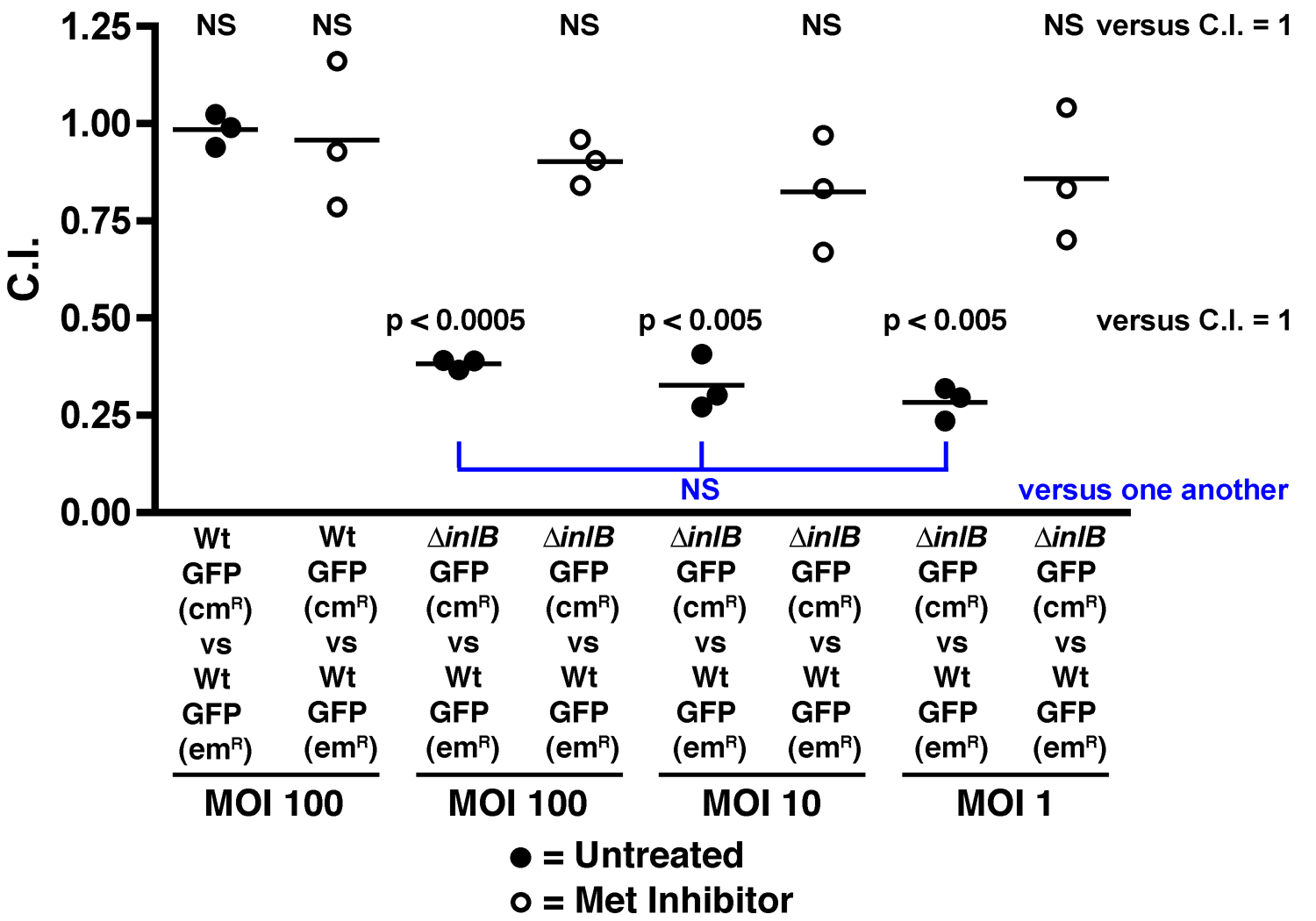

Supplement: Figure S3 — Expanded Figure 2F, InlB Promotes Invasion Local to the Bacterium. To determine whether the invasion defect of ΔinlB could be rescued, confluent MDCK monolayers were either untreated or treated with c-Met inhibitor prior and during infection with a 1∶1 ratio of WT∶WT, as a control, or WT∶ΔinlB at an MOI of 100∶1, 10∶1, or 1∶1 bacteria/cell. The ratio of the strains recovered, C.I., after gentamicin treatment was determined. (0.18 MB TIF) [file ppat.1000900.s003.tif]

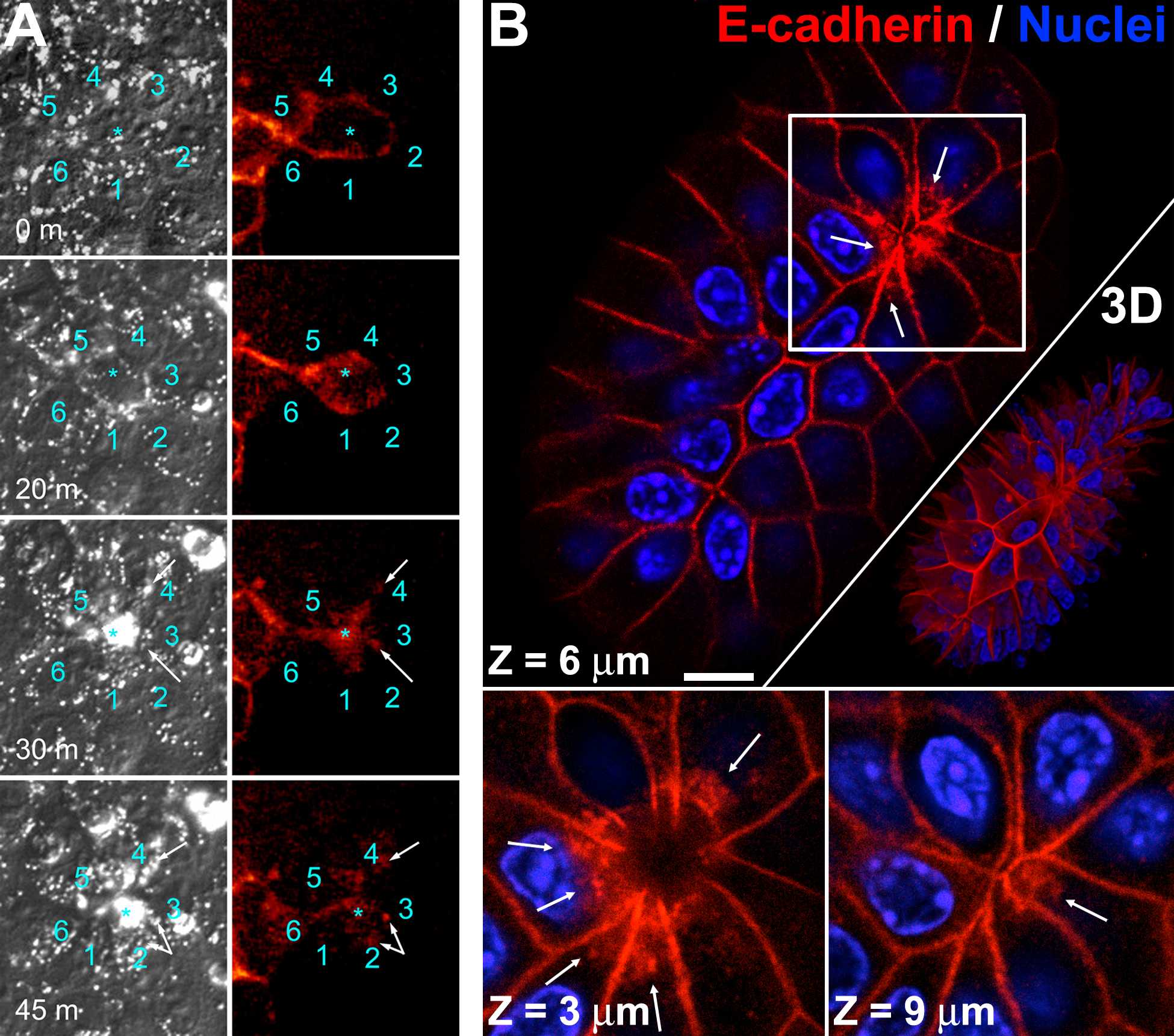

Supplement: Figure S4 — Endocytosis of E-cadherin at MCJs. (A) E-cadherin endocytosis during cell extrusion and MCJ formation. MDCK and MDCK E-cadherin-RFP cells were mixed and co-cultured to form a confluent monolayer for 1 day and then observed by DIC and fluorescence time-lapse microscopy. An extruding MDCK E-cadherin-RFP cell is marked with an asterisk and neighboring MDCK cells are numbered. Time in minutes, m, is indicated. Arrows indicate puncta of E-cadherin-RFP internalized by non-fluorescent neighboring cells during MCJ formation. (B) E-cadherin remodeling at an MCJ at the villus tip extrusion zone. Mouse intestinal tissue was stained with antibodies to E-cadherin, red, and with Topro-3 to visualize nuclei, blue, and imaged by 3D confocal microscopy. Depth from the apical cell surface, Z, is indicated. Arrows indicate intracellular puncta of E-cadherin. Scale bar 10 µm. (10.10 MB TIF) [file ppat.1000900.s004.tif]

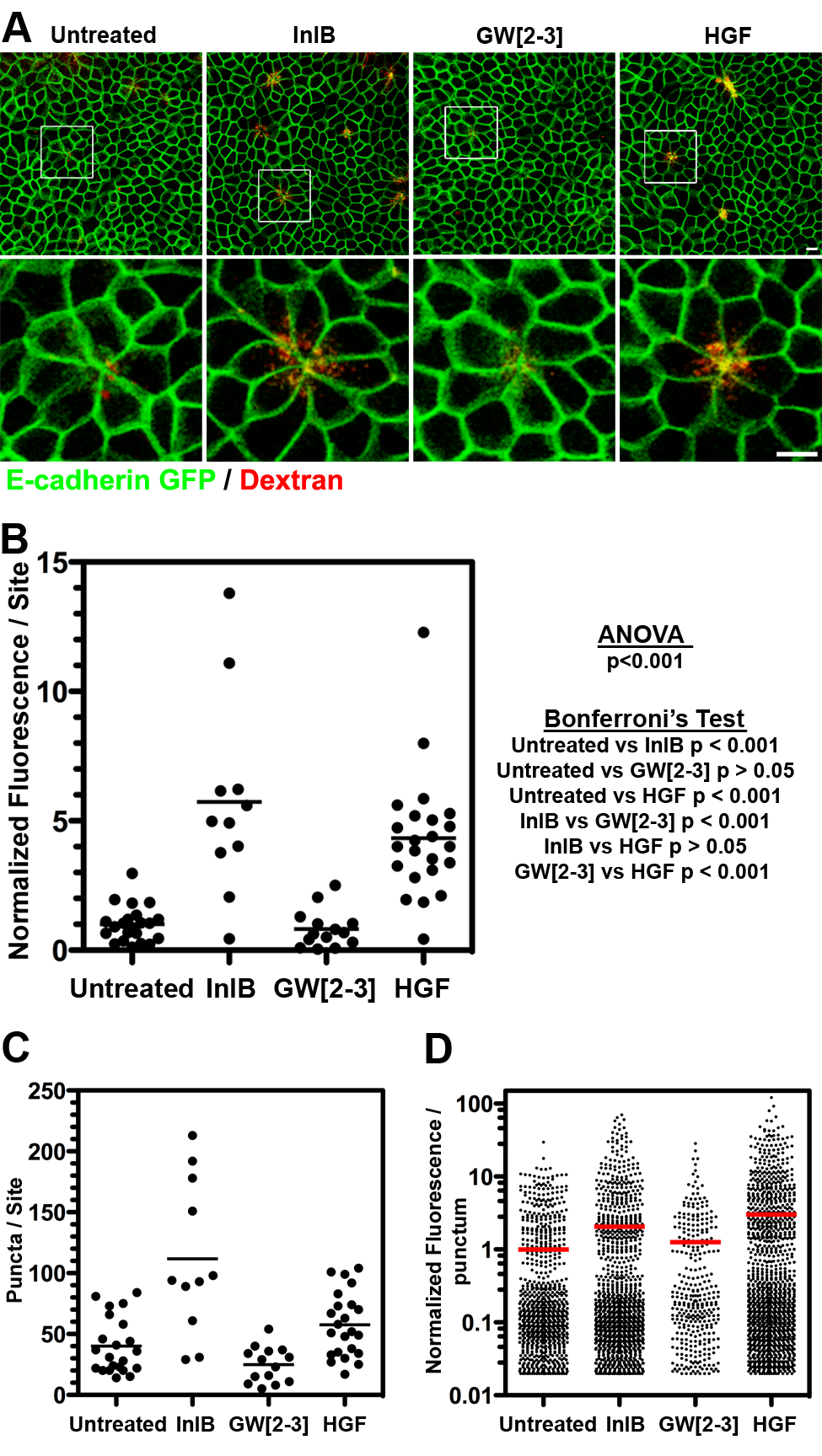

Supplement: Figure S5 — InlB and HGF, but not GW[2]–[3] Accelerate Endocytosis at MCJs. E-cadherin-GFP expressing MDCK monolayers were polarized on Transwell filters for 5 days and then treated with InlB, a truncated InlB containing only the C-terminal GW domains (GW[2]–[3]) or HGF for 1 h and then additionally treated with dextran, a fluid phase internalization marker, red, for 30 minutes. (A) 3D rendered views of polarized E-cadherin-GFP MDCK monolayers. Insets show multicellular junctions (MCJs). Scale bars 10 µm. (B) Quantification of dextran fluorescence in 50 µm×50 µm regions centered at multicellular junctions. (C) Quantification of dextran puncta in 50 µm×50 µm regions centered at MCJs. (D) Quantification of dextran fluorescence in all puncta analyzed at MCJs. (0.99 MB TIF) [file ppat.1000900.s005.tif]
